# Semen Analysis in Men with Testicular Cancer: Insights from a Large Fertility Preservation Cohort Toward Personalized Fertility Assessment

**DOI:** 10.3390/jpm16050263

**Published:** 2026-05-14

**Authors:** Federica Cariati, Maria Grazia Orsi, Anna Maione, Francesca Bagnulo, Raffaella Di Girolamo, Luigi Carbone, Alberto Servetto, Fabrizio Farina, Roberto Bianco, Sandro Cassiano Esteves, Carlo Alviggi, Alessandro Conforti

**Affiliations:** 1 Department of Public Health, School of Medicine, University of Naples Federico II, 80131 Naples, Italy; raffaella.digirolamo95@gmail.com (R.D.G.); alviggi@unina.it (C.A.); 2 Department of Pharmacy, School of Medicine, University of Naples Federico II, 80131 Naples, Italy; mariagrazia.orsi@unina.it; 3 Department of Neuroscience, Reproductive Science and Odontostomatology, University of Naples Federico II, 80131 Naples, Italy; rosymaio@hotmail.it; 4 Federico II of Naples Hospital, 80131 Naples, Italy; francesca.bagnulo@unina.it (F.B.); drcarboneluigi@gmail.com (L.C.); alessandro.conforti@unina.it (A.C.); 5 Department of Clinical Medicine and Surgery, University of Naples Federico II, 80131 Naples, Italy; alberto.servetto@unina.it (A.S.); roberto.bianco@unina.it (R.B.); 6 Department of Law, Economics, Management and Quantitative Methods, University of Sannio, Piazza Arechi II, 82100 Benevento, Italy; fafarina@unisannio.it; 7 ANDROFERT—Andrology and Human Reproduction Clinic, Campinas 13075-460, São Paulo, Brazil; s.esteves@androfert.com.br; 8 Division of Urology, Department of Surgery, Faculty of Medical Sciences, University of Campinas (UNICAMP), Campinas 13083-887, São Paulo, Brazil

**Keywords:** oncofertility, male fertility, testis cancer, fertility preservation, semen quality

## Abstract

**Background/Objectives**: Testicular cancer accounts for approximately 1% of all male malignancies, with an incidence ranging from 1 to 10 per 100,000 men and it predominantly affects young individuals, with nearly 60% of cases diagnosed between 15 and 35 years of age. In recent decades, the incidence of testicular cancer has markedly increased, paralleling a global rise in male infertility rates. Although chemotherapy is known to adversely affect fertility, the extent to which the tumor itself and its different histological subtypes impact semen quality remains incompletely understood. The aim of this study was to evaluate semen parameters in men diagnosed with testicular cancer prior to oncological treatment and to assess the possible association between tumor histology and semen quality. **Methods:** This retrospective study included data from 284 men diagnosed with testicular cancer who underwent semen cryopreservation prior to surgery, chemotherapy, or radiotherapy. Data were collected between January 2016 and June 2022 at the Maternal and Child Department of the University of Naples Federico II. Histopathological classification was available for 278 patients and revealed the following distribution: 59% (165/278) classic seminoma, 14.7% (41/278) seminomatous mixed germ cell tumors, 13.3% (37/278) non-seminomatous mixed germ cell tumors, and 12.6% (35/278) non-seminomatous germ cell tumors. **Results**: No significant association was observed between tumor histology and abnormal semen parameters. According to World Health Organization (WHO) reference values, semen parameters in patients with testicular cancer were predominantly distributed between the 5th and 25th percentiles. Microscopic semen analysis revealed significantly lower sperm concentration, total motility, and normal morphology in cancer patients (*p* < 0.001; *p* < 0.001; and *p* < 0.002, respectively). Logistic regression analysis showed a significant association between age and testicular cancer risk (*p* < 0.001), with a negative coefficient indicating that the likelihood of developing the disease decreases with increasing age. Additionally, patients with seminoma were significantly older than those with non-seminomatous tumors: on average, 4.07 years older than those with pure non-seminoma (*p* = 0.007) and 5.60 years older than those with mixed non-seminoma (*p* < 0.001). No statistically significant age differences were observed among non-seminomatous subtypes. **Conclusions**: These findings underscore the importance of systematic semen evaluation in young men diagnosed with testicular cancer and highlight the critical role of fertility preservation strategies in the comprehensive management of these patients.

## 1. Introduction

Testicular germ cell tumors (TGCTs) are relatively rare, with an incidence ranging from approximately 1/100,000 to 10/100,000. These tumors account for less than 1% of all malignancies in men but represent about 60% of cancers diagnosed in young males aged 15 to 40 years [1]. Epidemiological data indicate a sustained increase in the incidence of testicular cancer in recent decades [2]. Several conditions, including cryptorchidism, testicular dysgenesis, and infertility, are well-established risk factors for TGCT development [3,4,5]. The primary treatment strategies for TGCTs include surgery, chemotherapy, and radiotherapy [6,7]. While these modalities are highly effective in achieving cancer remission, they can significantly impact male fertility by adversely affecting semen parameters. The extent of chemotherapy-induced damage to spermatogenesis depends on the specific drugs used and the cumulative dose administered. The relationship between chemotherapy and infertility is multifaceted, involving both pathological and psychological factors. Some patients may have an inherent risk of subfertility even before tumor development, particularly in cases associated with testicular dysgenesis syndrome. Conversely, the tumor itself may negatively affect sperm quality and fertility even before treatment [8]. Chemotherapy regimens commonly used to treat TGCTs, such as those containing cisplatin, etoposide, and bleomycin, have been linked to an increased risk of infertility [6,9]. In addition, surgical interventions, including radical orchiectomy and retroperitoneal lymph node dissection, as well as chemotherapy and radiation therapy, can have significant adverse effects on reproductive function. While many of these effects are reversible, the timeframe for spermatogenesis recovery varies depending on the duration and type of treatment [10]. The management of young men affected by TGCTs who have not yet initiated family planning requires a thorough evaluation by multidisciplinary teams specialized in fertility preservation before the initiation of cancer treatment [11,12,13]. Semen cryopreservation is the most widely adopted strategy to preserve fertility in these patients, as also reported in other oncological settings, including hematological malignancies [14]. However, limited data are available regarding the overall quality of semen in men with TGCTs and whether different histological subtypes influence semen parameters. Despite the well-documented impact of oncological treatments on male fertility, increasing evidence suggests that semen impairment may already be present at the time of diagnosis in patients with testicular germ cell tumors (TGCTs). This observation supports the hypothesis that TGCTs and male infertility may share common pathogenic mechanisms, including testicular dysgenesis, hormonal imbalance, and genetic susceptibility. Within this framework, a personalized medicine approach becomes particularly relevant, as individual variability in tumor biology and reproductive function may influence both disease presentation and fertility outcomes. However, the extent to which different histopathological subtypes contribute to pre-treatment semen impairment remains unclear, highlighting the need for individualized risk assessment models. This retrospective study aimed to analyze the semen parameters of men with TGCTs undergoing cryopreservation and compare them with those of healthy men seeking in vitro fertilization (IVF) due to female factor infertility. Furthermore, we assessed whether semen quality in patients with TGCTs was influenced by tumor histology.

## 2. Materials and Methods

### 2.1. Study Population

This study included men diagnosed with testicular germ cell tumors (TGCTs) who were referred for fertility preservation at the Fertility Preservation Unit of the University of Naples Federico II between January 2016 and June 2022. The primary outcome was the evaluation of semen parameters according to the World Health Organization (WHO) 2021 guidelines, with age and histological tumor type included as variables of interest. To establish an internal control group from the same geographic region, semen analysis results were collected from 51 male partners of couples undergoing in vitro fertilization (IVF) due to female factor infertility (bilateral tubal obstruction or oocyte donation), with no history of cancer. All control subjects underwent urological evaluation prior to inclusion to exclude male infertility factors. All participants provided written informed consent, and the study protocol was approved by the institutional review board. Semen analyses were conducted following standardized WHO 2021 procedures, with trained laboratory personnel and established internal quality control measures. In addition to conventional semen parameters, patient-specific variables, including age and tumor histology, were systematically recorded to enable an individualized analytical approach. Statistical analyses were designed to assess both group differences and interindividual variability, supporting more personalized risk stratification in TGCT patients.

### 2.2. Sperm Cryopreservation

Semen collection and cryopreservation were performed pre-operatively and prior to any gonadotoxic treatment. No post-operative semen analyses were included in this study. To minimize the risk of potential infection transmission, all patients underwent pre-screening for hepatitis B and C, HIV, and syphilis before semen cryopreservation. Semen samples were collected via masturbation after 2–3 days of sexual abstinence and analyzed manually in duplicate according to the WHO 2021 manual. After collection and liquefaction, semen specimens were cryopreserved as raw samples following the manufacturer’s protocol. A cryoprotective medium containing TEST-yolk buffer was added dropwise at room temperature over 30 s until a 1:1 ratio of semen to buffer was reached. The mixture was kept at room temperature for 10 min before being transferred into patient-labeled cryovials. Samples were initially exposed to liquid nitrogen vapor for 30 min and then stored in liquid nitrogen at −196 °C. Patients whose semen volume was below the WHO reference values were advised to provide additional samples to ensure that at least six cryovials were preserved.

### 2.3. Statistical Analysis

To compare patients with testicular cancer to the control group, an independent samples *t*-test was performed to assess differences between the two unrelated groups. The null hypothesis stated that there was no significant difference between the group means, whereas the alternative hypothesis proposed a statistically significant difference. Before conducting parametric tests, the data were examined to ensure that the assumptions required for an independent *t*-test were met (i.e., absence of outliers, normality assessed using the Shapiro–Wilk test, and homogeneity of variances evaluated with Levene’s test). When the *p*-value for Levene’s test was greater than 0.05, equal variances were assumed. If variances differed significantly (e.g., for semen volume or sperm concentration), a nonparametric Mann–Whitney U test was applied. Additionally, a logistic regression model was used to assess whether age significantly influences the likelihood of developing testicular cancer, and a one-way analysis of variance (ANOVA) was used to compare the mean values of multiple independent groups and to determine whether significant differences existed among the different histological subtypes of testicular cancer. All statistical analyses were performed using SPSS version 22.0 for Windows (Statistical Package for the Social Sciences, IBM Corp., Armonk, NY, USA). A *p*-value < 0.05 was considered statistically significant.

## 3. Results

**Figure 1:**
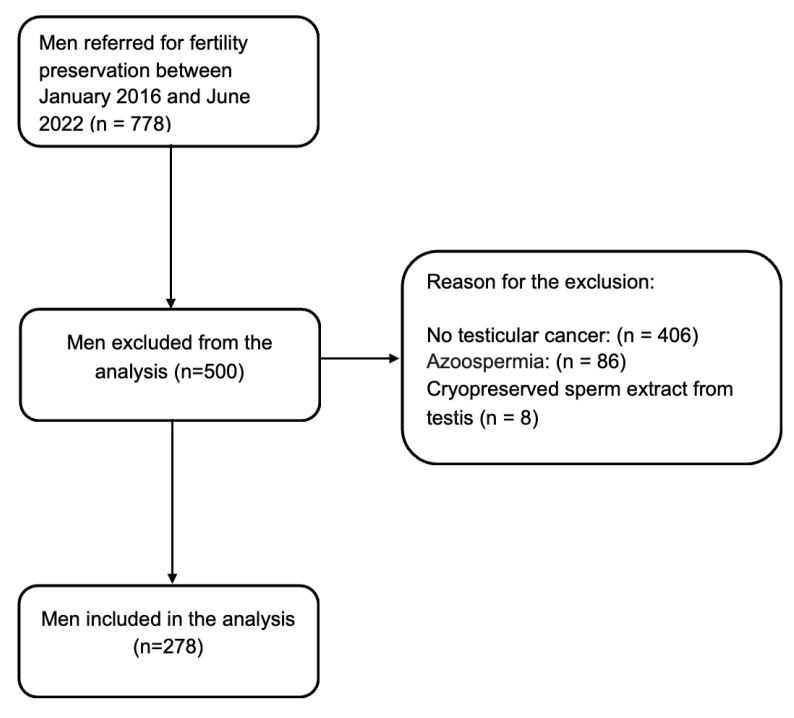
Flow chart.

Out of 778 men referred to our center for fertility preservation, 278 patients diagnosed with testicular germ cell tumors (TGCTs) who underwent semen cryopreservation were included in the analysis (Figure 1). As shown in Figure 1, patients without testicular cancer, those in whom cryopreservation was not performed due to azoospermia (i.e., severe impairment of sperm parameters), and those who underwent cryopreservation of testicular sperm were excluded from the analysis.

**Figure 2:**
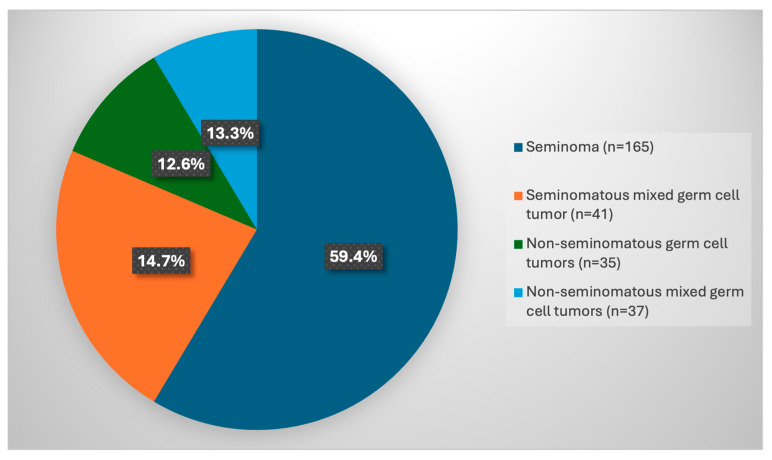
Histological classification of testicular tumor.

Based on the WHO histopathological classification [15], classic seminoma was the most common histological type, accounting for 59% (165/278) of cases. Seminomatous mixed germ cell tumors (SMMGTs) were observed in 14.7% (41/278) of patients, while 13.3% (37/278) had non-seminomatous mixed germ cell tumors (NSMGTs). In addition, 12.6% (35/278) of cases were classified as non-seminomatous germ cell tumors (NSGCTs), including five cases of teratoma, 28 cases of embryonal carcinoma, and two cases of choriocarcinoma (Figure 2). The logistic regression model aims to assess whether age significantly influences the likelihood of developing testicular cancer (Table 1). The *p* value (<0.001) highlights a non-random relationship between age and diagnosis. The negative coefficient explains how the likelihood of developing testicular cancer decreases as age increases. Specifically, for each additional year of age, the odds of developing the tumor decrease by approximately 14.5%. In addition, the ANOVA analysis completes the clinical picture, shifting the focus from “overall risk” to the age difference between the different specific types of testicular cancer (Table 2). While logistic regression indicated that younger people were at greater risk, ANOVA reveals that even within cancer cases, the mean age varies significantly depending on the histotype. The *p*-value is significant (*p* < 0.001), indicating that the mean age is not the same for the four tumor types considered. There is at least one pair of tumor types that has a statistically significant difference in mean age. Seminoma vs. Non-Seminoma: Seminoma occurs in significantly older patients than non-seminoma types. Compared to pure non-seminoma, patients with seminoma are on average 4.07 years older (*p* = 0.007). Compared to mixed non-seminoma, patients with seminoma are on average 5.60 years older (*p* < 0.001). No statistically significant age differences emerge between the different types of non-seminoma or between these groups and mixed non-seminomatous tumors.

According to the WHO 2021 semen analysis criteria, most semen parameters in patients with TGCTs fell between the 5th and 25th percentiles, as shown in Table 3.

Semen volume was the only parameter above the 50th percentile. When semen characteristics were analyzed according to histopathological classification, no statistically significant differences were observed among tumor subtypes (Table 4). A comparison of semen parameters between patients with testicular germ cell tumors (TGCTs) and the control group—comprising 51 male partners of couples undergoing in vitro fertilization (IVF) due to female factor infertility—revealed several significant differences. Sperm concentration, total motility, and the percentage of spermatozoa with normal morphology were significantly lower in patients with testicular cancer (*p* < 0.05) (Table 5). However, no significant differences in semen parameters were found among the different histological subtypes of TGCTs.

## 4. Discussion

Our study confirmed that classic seminoma is the most common histological subtype of testicular germ cell tumors. Moreover, the logistic regression model supports a well-established clinical observation: testicular cancer predominantly affects young individuals, with the probability of developing the disease decreasing as age increases. Although testicular cancer is generally considered a disease of young adults, our findings further highlight age-related differences among histological subtypes. Classic seminomas tend to occur in slightly older patients (typically between 30 and 40 years), whereas non-seminomatous tumors are more frequently diagnosed in younger individuals (usually between 20 and 30 years). Accordingly, very young patients are more likely to present with non-seminomatous tumors, while the probability of classic seminoma increases with advancing age. Our results demonstrate that men affected by testicular germ cell tumors exhibit significantly impaired semen parameters compared to those undergoing in vitro fertilization due to female factor infertility alone. Importantly, this impairment appears to be independent of histopathological subtype, suggesting that the tumor itself, rather than its classification, is the main determinant of semen quality. This single-center study represents one of the most extensive investigations of semen parameters in men diagnosed with TGCTs. The increasing number of patients seeking sperm cryopreservation at our center between 2015 and 2022 reflects a growing awareness of fertility preservation, consistent with recent epidemiological trends [16]. Previous studies have similarly reported impaired semen parameters in men with TGCT [17,18,19], consistently showing reductions in sperm concentration, motility, and morphology. However, in contrast to earlier reports [1,20] suggesting greater impairment in non-seminomatous tumors, our findings did not reveal significant differences between histological subtypes. This discrepancy may be explained by the larger sample size of our cohort and by the distinction between pure and mixed histological forms, which was not consistently addressed in previous studies. Several mechanisms may underlie the observed impairment in semen quality among TGCT patients. Dias et al. identified altered protein expression in seminoma patients, associated with spermatogenic dysfunction, reduced sperm kinematics and motility, and impaired fertilization capacity [21]. Additionally, metabolic alterations in ATP production pathways have been reported [22], indicating a shift from oxidative phosphorylation to glycolysis and resulting in mitochondrial dysfunction. Increased oxidative stress has also been described in TGCT patients [23,24]. Together, these factors contribute to sperm dysfunction and may further compromise fertilization potential. Excessive oxidative stress is also known to negatively affect reproductive outcomes, including natural conception and IVF success rates [25,26]. Therefore, targeted strategies aimed at reducing oxidative stress may represent a promising approach to improve fertility outcomes in this population. In this context, advanced sperm selection techniques designed to minimize oxidative stress-induced damage have been proposed as potential adjunctive tools in assisted reproduction [27]. The seminal microbiome and genomic integrity have recently emerged as potential modulators of reproductive function and exhibit considerable interindividual variability [28,29,30,31,32]. Notably, men with TGCTs appear to harbor a distinct seminal microbiome compared to healthy controls, suggesting that specific microbial profiles may contribute to tumor-associated reproductive impairment [33]. The clinical relevance of our findings underscores the importance of early and systematic fertility preservation in men diagnosed with TGCTs. International guidelines consistently recommend sperm cryopreservation prior to the initiation of cancer treatment [34,35,36,37,38], and the integration of fertility counseling into oncological care is essential to inform patients about potential reproductive risks and available preservation strategies [39,40]. The success rate of assisted reproductive technologies using cryopreserved sperm from cancer patients ranges from 33% to 56%, with no significant differences in neonatal outcomes compared to the general population. Moreover, although sperm motility parameters decline after cryopreservation, no significant differences have been observed between cancer patients and fertile men [41]. Given the established association between male infertility and an increased risk of malignancy, infertile men should undergo careful evaluation to identify potential underlying conditions, including testicular cancer [42,43]. Within the framework of personalized medicine, these findings have important clinical implications. The identification of impaired semen parameters at diagnosis supports the need for individualized fertility counseling and early implementation of sperm cryopreservation. Furthermore, the integration of clinical and biological patient-specific factors may improve the prediction of reproductive outcomes and support tailored management strategies. In the future, personalized approaches may guide not only fertility preservation but also targeted interventions aimed at mitigating treatment-related gonadotoxicity and optimizing reproductive potential in men with TGCTs.

## 5. Conclusions

In conclusion, this study confirms that men diagnosed with testicular germ cell tumors (TGCTs) exhibit significantly impaired semen quality. However, histopathological classification, including mixed tumor forms, does not appear to significantly influence semen parameters. These findings underscore the importance of timely fertility preservation strategies in affected men. Further research is required to validate these results and elucidate the underlying pathophysiological mechanisms contributing to sperm dysfunction in patients with TGCTs.

### Limitations and Future Research

A limitation of the present study is the lack of data on potential confounding factors such as hormone levels (FSH, LH, testosterone) and lifestyle variables (smoking status, BMI), which prevented the use of multivariate regression analyses. Therefore, the results should be interpreted with caution, as these unmeasured variables may have influenced semen quality. Future research should focus on developing personalized predictive models integrating clinical, hormonal, genetic, and molecular data to better characterize fertility impairment in TGCT patients. Prospective multicenter studies with standardized methodologies are needed to validate these findings and enhance their generalizability. In particular, the integration of advanced biomarkers, oxidative stress profiling, and seminal microbiome characterization may enable more precise identification of patients at higher risk of infertility, ultimately supporting tailored fertility preservation strategies and individualized therapeutic interventions.

## Figures and Tables

**Table 1:** Logistic model.

Logistic Model						
	B	S.E.	Wald	Gl	Sign.	Exp(B)
Age	−0.157	0.026	37.485	1	0.000	0.855
Constant	7.111	0.951	55.867	1	0.000	1225.311

Variables included in phase 1: Age.

**Table 2:** Anova analysis.

ANOVA						
Age						
	SS	df		MS	F	Sign.
Between	1251.645	3		417.215	9.473	0.000
Within	12,023.150	273		44.041		
Total	13,274.794	276				
Multiple comparisons						
		Mean Difference (I–J)	Standard Error	Significance	95% Confidence Interval	
					Lower Bound	Upper Bound
Seminoma	Seminomatous mixed germ cell tumors	2.57694	1.15805	0.161	−0.5007	5.6546
	Non-seminomatous germ cell tumors	4.06753 *	1.23500	0.007	0.7854	7.3497
	Non seminomatous mixed germ cell tumors	5.60404 *	1.22077	0.000	2.3597	8.8484
Seminoma mixed germ cell tumors	Seminoma	−2.57694	1.15805	0.161	−5.6546	0.5007
	Non-seminomatous germ cell tumors	1.49059	1.52724	1.000	−2.5682	5.5494
	Non-seminomatous mixed cell tumors	3.02710	1.51576	0.281	−1.0012	7.0554
Non-seminomatous germ cell tumors	Seminoma	−4.06753 *	1.23500	0.007	−7.3497	−0.7854
	Seminoma mixed germ cell tumors	−1.49059	1.52724	1.000	−5.5494	2.5682
	Non seminomatous mixed germ cell tumors	1.53651	1.57533	1.000	−2.6501	5.7231
Non-seminomatous mixed germ cell tumors	Seminoma	−5.60404 *	1.22077	0.000	−8.8484	−2.3597
	Seminoma mixed germ cell tumors	−3.02710	1.51576	0.281	−7.0554	1.0012
	Non-seminomatous germ cell tumors	−1.53651	1.57533	1.000	−5.7231	2.6501

* The mean difference is significant at the 0.05 level.

**Table 3:** Results of semen analysis in patients with testicular cancer compared to percentile values (5th, 25th, 50th, 75th, 95th) of WHO 2021.

	Patients with Testicular Cancer (*n* = 278)	5th Percentile	25th Percentile	50th Percentile	75th Percentile	95th Percentile
Volume (mL)	3.2 ± 1.6	1.4	2.3	3.0	4.2	6.2
Concentration (×10^6^/mL)	18.9 ± 23.3	16	36	66	110	208
Progressive motility (%) (a + b)	31.3 ± 17.4	30	45	55	63	77
Total motility (%) (a + b + c)	46.0 ± 19.3	42	55	64	73	90
Normal morphology; Kruger criteria (%)	4.1 ± 2.8	4	8	14	23	39

**Table 4:** Results of semen analysis in patients with testicular cancer based on histopathological classification.

	Seminoma (*n* = 165)	SMMX (*n* = 41)	NSGCT (*n* = 35)	NSMGCT (*n* = 37)	F-Statistic	*p*-Value
Age	32.3 ± 6.5	29.8 ± 6.1	28.3 ± 6.7	26.7 ± 7.3		
Volume (mL)	3.3 ± 1.7	3.6 ± 1.5	3.1 ± 1.4	3.1 ± 1.4	0.905	0.439
Concentration (×10^6^/mL)	21.3 ± 26.3	13.5 ± 18.9	14.7 ± 17.4	18.0 ± 17.8	0.601	0.615
Rapid progressive motility (%)	1.4 ± 3.0	1.0 ± 2.4	1.0 ± 2.0	1.2 ± 2.8	0.238	0.870
Progressive motility (%) (a + b)	31.6 ± 17.8	28.9 ± 17.1	30.9 ± 19.5	31.5 ± 14.6	0.318	0.812
Total motility (%) (a + b + c)	45.9 ± 19.6	43.3 ± 18.9	45.8 ± 22.9	47.9 ± 15.6	0.358	0.784
Normal morphology (%)	4.1 ± 2.6	3.3 ± 3.3	4.3 ± 2.7	4.7 ± 3.2	1.461	0.226

**Table 5:** Results of semen analysis in patients with testicular cancer compared to the control group.

	Testicular Cancer (*n* = 278)	Control Group (*n* = 51)	*p*-Value
Age	30.2 ± 6.9	38.0 ± 5.8	<0.001
Volume (mL)	3.2 ± 1.6	2.8 ± 1.1	0.051
Concentration (×10^6^/mL)	18.9 ± 23.3	53.2 ± 41.0	<0.001
Rapid progressive motility (%)	1.3 ± 2.8	4.2 ± 4.8	<0.001
Progressive motility (%) (a + b)	31.3 ± 17.4	41.9 ± 14.8	<0.001
Total motility (%) (a + b + c)	46.0 ± 19.3	56.2 ± 14.7	<0.001
Normal morphology (%)	4.1 ± 2.8	5.4 ± 2.4	0.002

## Data Availability

The data presented in this study are available on reasonable request from the corresponding author. The data are not publicly available due to privacy and ethical restrictions.

## Abbreviations

The following abbreviations are used in this manuscript:

WHO	World Health Organization
TGCT	Testicular germ cell tumor
IVF	In vitro fertilization
SMMX	Seminomatous mixed germ cell tumor
NSGCT	Non-seminomatous germ cell tumor
NSMGCT	Nonseminomatous mixed germ cell tumor
